# Case Report: Secukinumab induced pustular eruption in a patient with ankylosing spondylitis

**DOI:** 10.3389/fmed.2026.1786722

**Published:** 2026-03-13

**Authors:** Zenan Tang, Xiaoyang Liu

**Affiliations:** Department of Dermatology, Peking University People's Hospital, Beijing, China

**Keywords:** adverse drug reaction, ankylosing spondylitis, case report, JAK inhibitor, paradoxical reaction, pustular eruption, secukinumab

## Abstract

We report a case of a 33-year-old male with ankylosing spondylitis (AS) who had been receiving secukinumab (150 mg monthly) for 2 years. Approximately 1 year after initiating treatment, the patient developed recurrent, multiple sterile pustules on the palms and soles. Various topical treatments, including corticosteroids and calcipotriol, yielded minimal improvement. 1 week prior to presentation, new pustular lesions emerged on the trunk and extremities, accompanied by pruritus followed by burning sensations and tenderness. Histopathological examination revealed subcorneal pustule formation within the epidermis and sparse lymphocytic infiltration in the dermis. A diagnosis of secukinumab-induced pustular reaction was made. Following the discontinuation of secukinumab and the initiation of upadacitinib (15 mg/day) for over 1 month, the pustules largely resolved, and symptoms were significantly alleviated. This case suggests that IL-17A inhibitors may trigger pustular cutaneous reactions in AS patients. Clinicians should remain vigilant regarding such adverse effects and adjust treatment regimens promptly. JAK inhibitors may provide an effective therapeutic option for managing these drug-induced reactions.

## 1 Introduction

Secukinumab, a monoclonal antibody targeting interleukin-17A (IL-17A), plays a pivotal role in modern therapeutics, particularly for moderate-to-severe plaque psoriasis, psoriatic arthritis, and ankylosing spondylitis (AS). Compared to traditional therapies, secukinumab significantly improves clinical symptoms and patient quality of life (1, 2). Specifically, in the field of ankylosing spondylitis, Secukinumab has been approved by the FDA for the treatment of active radiographic axial spondyloarthritis and active non-radiographic axial spondyloarthritis (nr-axSpA) (3, 4). However, atypical cutaneous adverse reactions have been reported during secukinumab treatment (5). Although relatively rare, these reactions pose new clinical challenges. Herein, we report a case of AS involving multiple pustular eruptions on the palms, soles, and trunk during secukinumab therapy. This case is noteworthy as the patient had no prior personal or family history of psoriasis. Furthermore, the clinical presentation was characterized by a lack of typical erythematous scales associated with palmoplantar pustulosis, distinguishing it from previously reported cases. This case provides new insights for clinicians regarding the potential cutaneous side effects of secukinumab and underscores the importance of vigilance during its administration.

## 2 Case presentation

A 33-year-old male presented with a 1-year history of palmoplantar pustules and a 1-week history of new-onset pustules on the trunk and extremities, accompanied by pruritus, burning, and tenderness. The patient had been diagnosed with AS 4 years ago; previous treatments with celecoxib and sulfasalazine failed to adequately control back pain and peripheral joint symptoms. Secukinumab therapy was initiated 2 years ago at a dose of 150 mg subcutaneously every month, which was later adjusted to every 2–3 months after achieving disease stability.

One year ago, the patient developed skin lesions without an obvious trigger, presenting as multiple, symmetrically distributed, pea-sized sterile pustules on the palms and soles, some of which coalesced. The rash initially presented with pruritus, followed by burning and tenderness. Topical corticosteroids and calcipotriol were ineffective. Over the last 6 months, the patient also developed “oil-drop” discoloration and distal onycholysis of the finger and toe nails. In the past week, the rash worsened with new pustules appearing on the trunk and limbs. The patient received the last subcutaneous injection of 150 mg secukinumab approximately 2 months ago and presented to the dermatology outpatient clinic for an evaluation regarding the continuation of treatment. He denied any family history of psoriasis or drug allergies, and his hepatic and renal functions were within normal limits.

### 2.1 Physical examination

**Figure 1:**
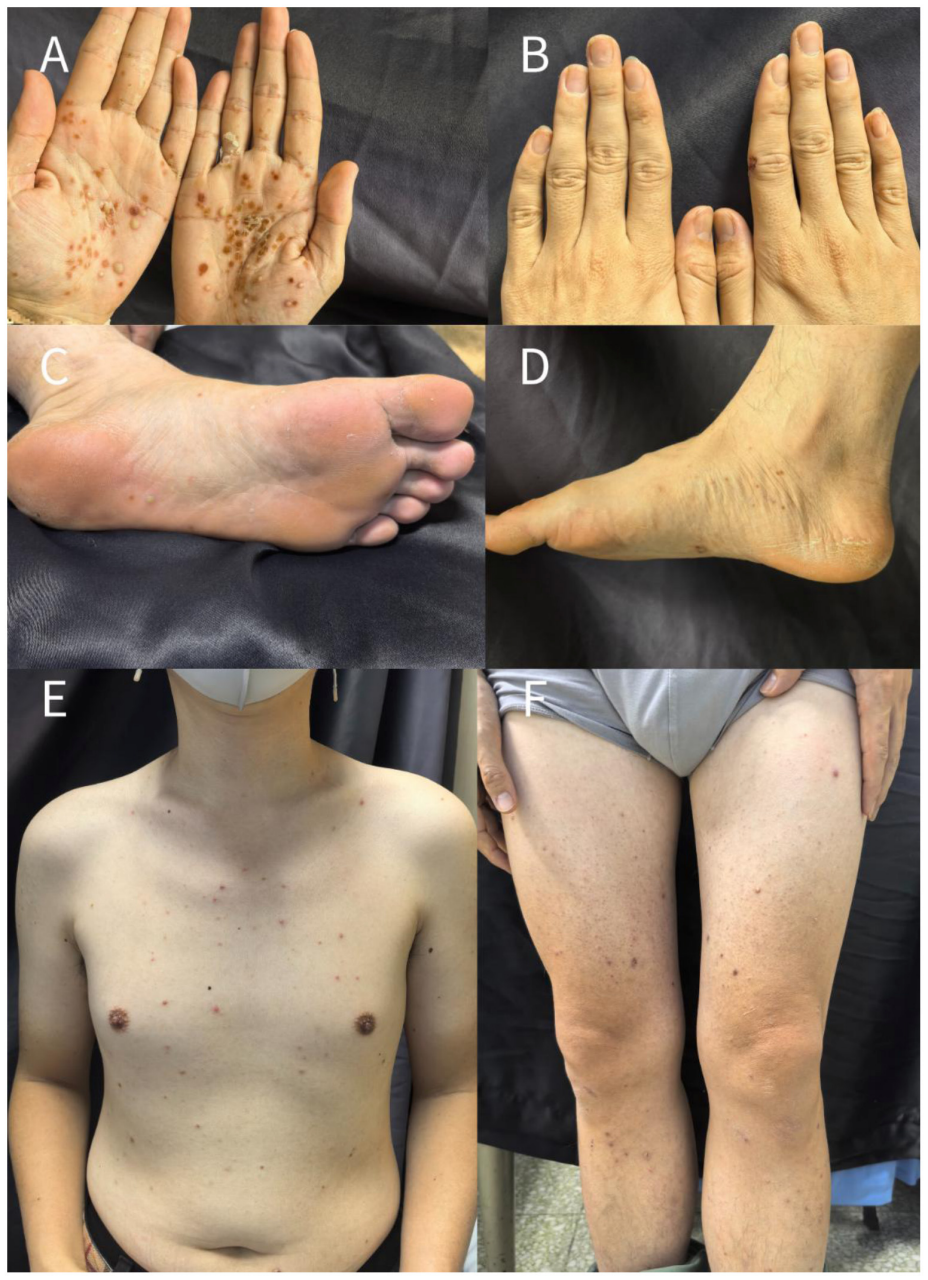
Scattered pustules were observed on the palms, soles, and trunk, with some lesions coalescing or presenting as dried crusts. **(A)** Palm; **(B)** Dorsum of the hand; **(C)** Left foot; **(D)** Right foot; **(E)** Trunk; **(F)** Both lower limbs.

Scattered pustules were observed on the palms, soles, and trunk, with some lesions coalescing or presenting as dried crusts. Distal onycholysis and “oil-drop” signs were noted on several nail plates (Figure 1).

### 2.2 Laboratory investigations

Routine blood tests, Antistreptolysin O, Cytokine test (IL1β,2,4,5,6,8,10,22,17A,17F,TNF-α), coagulation profiles, and serological screening for HIV, hepatitis B, and syphilis were all unremarkable.

### 2.3 Histopathology

**Figure 2:**
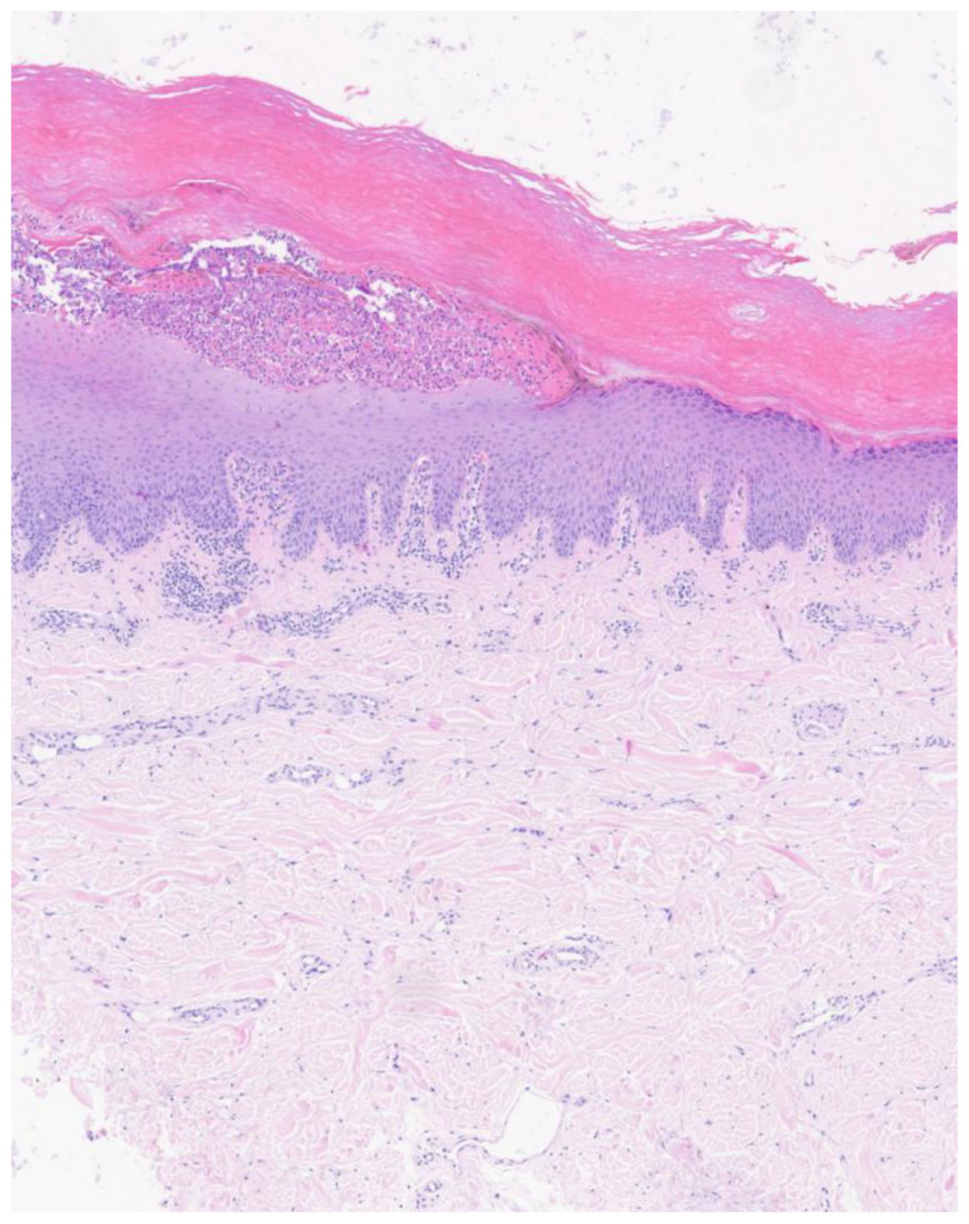
A skin biopsy showed focal parakeratosis, subcorneal pustule formation within the epidermis, and mild lymphocytic infiltration in the upper dermis.

A skin biopsy showed focal parakeratosis, subcorneal pustule formation within the epidermis, and mild lymphocytic infiltration in the upper dermis (Figure 2).

### 2.4 Diagnosis

Secukinumab induced pustular reaction.

### 2.5 Treatment

Secukinumab was discontinued, and oral upadacitinib (15 mg daily) was initiated.

### 2.6 Follow-up

**Figure 3:**
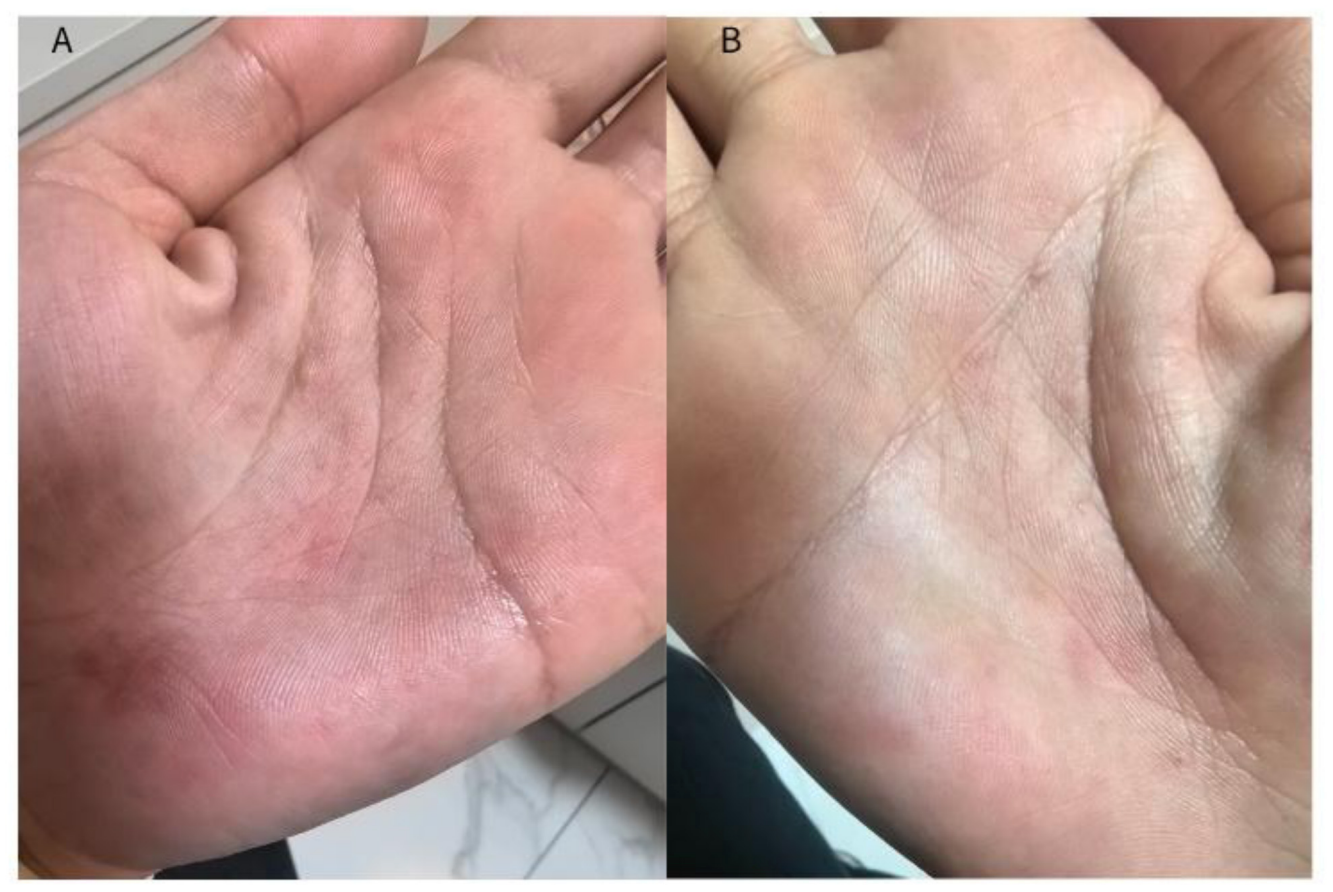
After one month of treatment, the symptoms significantly improved, and the majority of the pustules had resolved. **(A)** Left hand; **(B)** Right hand.

After 1 month of treatment, the symptoms significantly improved, and the majority of the pustules had resolved (Figure 3).

## 3 Discussion

In recent years, IL-17A inhibitors have gained widespread use as targeted biological agents for chronic inflammatory diseases such as moderate-to-severe plaque psoriasis, psoriatic arthritis, and AS. By specifically inhibiting the IL-17A cytokine, these agents have markedly improved outcomes for patients refractory to traditional therapies, with their efficacy and safety confirmed by extensive clinical trials and real-world data (6). However, as their clinical application expands, reports of associated adverse effects have increased. Although cutaneous adverse reactions are relatively infrequent, they are phenotypically diverse, including eczematous rashes, urticaria, folliculitis, and vitiligo. Pustular cutaneous reactions, in particular, have emerged as a specific concern (7).

Literature has previously documented that IL-17A inhibitors may induce palmoplantar pustulosis in patients being treated for plaque psoriasis. This phenomenon, often termed a “paradoxical reaction,” is thought to result from an imbalance in the local immune microenvironment following the inhibition of the IL-17A pathway (8). IL-17A is essential for maintaining skin barrier function and anti-infective immunity; blocking its activity may lead to abnormal keratinocyte proliferation and inflammatory cell infiltration, thereby triggering pustule formation (8). Notably, such reactions are rarely reported in AS patients receiving IL-17A inhibitors. In this case, a formal causality assessment strongly supports the diagnosis of a secukinumab-induced paradoxical reaction manifesting as pustular eruption. Primarily, a clear temporal relationship was observed: the patient developed sterile pustules approximately 1 year after initiating secukinumab, with symptoms progressively exacerbating as the duration of drug exposure increased. Secondly, infectious etiologies were systematically excluded, as laboratory investigations for antistreptolysin O and serological screenings for HIV, hepatitis B, and syphilis were all unremarkable. Furthermore, the patient exhibited significant clinical improvement following drug withdrawal (dechallenge); within 1 month of discontinuing secukinumab and switching to upadacitinib, the cutaneous lesions and pruritus had largely resolved.

Palmoplantar pustulosis is a recognized but rare paradoxical reaction in patients with AS undergoing biologic therapy. While this phenomenon is more commonly associated with TNF-α inhibitors, paradoxical pustular reactions triggered by IL-17A inhibitors are exceedingly rare (9). Alnaqbi et al. previously reported a case of a 45-year-old male with AS who was initially treated with infliximab for 4 years with significant efficacy, followed by a gradual loss of response. Upon switching to secukinumab, the patient experienced a worsening of arthritis symptoms and the onset of palmoplantar pustules after only five injections (10). In contrast to that case, our patient had no prior history of TNF inhibitor exposure, further emphasizing the direct role of IL-17A inhibition in triggering this adverse event.

The adjustment of dosing intervals for biologics is a widely adopted approach in real-world clinical practice and is increasingly being recommended in treatment guidelines by professional scientific societies (11). However, previous studies on extended Secukinumab dosing in psoriasis have not reported an increase in adverse events (AEs) (12–15). Nevertheless, previous studies have suggested that interval prolongation may theoretically lead to lower trough drug concentrations and potentially increase immunogenicity, including the development of anti-drug antibodies (ADA), which might compromise therapeutic efficacy (16). In our patient, however, there was no clinical evidence of secondary loss of response to secukinumab, as ankylosing spondylitis symptoms remained stable even after the dosing interval was extended. Moreover, dose spacing is sometimes considered a potential strategy to mitigate biologic-related adverse reactions. In this case, despite the dosing frequency was reduced to every 2–3 months due to stable rheumatologic disease activity, the pustular eruption did not improve; instead, it persisted and progressively worsened with new lesions continuing to develop. These observations suggest that interval prolongation was unlikely to be the primary provoking factor, and that the paradoxical reaction was more likely related to IL-17A inhibition itself, ultimately requiring drug discontinuation.

This case expands the known spectrum of adverse drug reactions for this class of biologics, suggesting that IL-17A inhibitors can induce pustular reactions regardless of the primary indication. Furthermore, the lack of typical erythema and scaling in this patient highlights the clinical heterogeneity of IL-17-induced pustular reactions, necessitating further research into the underlying mechanisms.

Regarding therapeutic management, given the clear temporal correlation between secukinumab and the pustular reaction, the primary step was the discontinuation of the drug to remove the persistent immune stimulus. TNF inhibitors are indeed a primary therapeutic option for patients with ankylosing spondylitis (17). While some meta-analyses indicate that the efficacy of monoclonal antibodies against TNF may surpass that of Secukinumab, safety data suggest that Secukinumab carries a lower risk of adverse events and serious adverse events during treatment (18). Furthermore, evidence indicates that TNF inhibitors are themselves among the most frequent types of biologics associated with the induction of paradoxical psoriasis or pustulosis of the palms and soles (9). Given that this patient had already developed a clear case of paradoxical pustular rash related to biologic therapy, we considered during clinical decision making that switching to another class of biologics, such as a TNF inhibitor, might still carry a potential risk of triggering a similar paradoxical reaction. Unlike their role in treating plaque psoriasis, the efficacy of anti TNF agents for pustulosis is often considered questionable (19). To address the cutaneous symptoms, particularly the severe pruritus, we initiated upadacitinib, a JAK1 inhibitor. Upadacitinib is an oral, selective JAK1 inhibitor, modulating the JAK/STAT pathway to dampen multiple pro-inflammatory cytokine signals. While approved for conditions like psoriatic arthritis, atopic dermatitis (20, 21). For ankylosing spondylitis, upadacitinib has been approved by the FDA for the treatment of adults with active ankylosing spondylitis who have had an inadequate response or intolerance to TNF inhibitors (3). Additionally, it is indicated for the treatment of adults with active non-radiographic axial spondyloarthritis with objective signs of inflammation [elevated C-reactive protein (CRP) and/or abnormal findings on magnetic resonance imaging (MRI)] who have had an inadequate response to nonsteroidal anti-inflammatory drugs (NSAIDs) (3). However, the use of upadacitinib for pustular skin lesions induced by secukinumab in the treatment of AS remains off-label. Several studies and case reports have demonstrated that JAK inhibitors (such as upadacitinib and tofacitinib) are effective for palmoplantar pustulosis, providing rapid relief of pustules, itching, and pain (22). In this case, the significant resolution of pustules and pruritus within 1 month of starting upadacitinib (15 mg/day) validates the therapeutic value of JAK inhibitors in managing IL-17A inhibitor-induced pustular reactions. This provides a practical reference for the management of similar clinical cases.

## Data Availability

The raw data supporting the conclusions of this article will be made available by the authors, without undue reservation.
